# Direct Cloning and Heterologous Expression of the Dmxorosin Biosynthetic Gene Cluster from *Streptomyces thermolilacinus* SPC6, a Halotolerant Actinomycete Isolated from the Desert in China

**DOI:** 10.3390/ijms26041492

**Published:** 2025-02-11

**Authors:** Maoxing Dong, Huyuan Feng, Wei Zhang, Wei Ding

**Affiliations:** 1 School of Life Sciences, Lanzhou University, Lanzhou 730000, China; dongmx17@lzu.edu.cn (M.D.); fenghy@lzu.edu.cn (H.F.); 2 Key Laboratory of Extreme Environmental Microbial Resources and Engineering of Gansu Province, Northwest Institute of Eco-Environment and Resources, Chinese Academy of Sciences, Lanzhou 730000, China; bestdw@163.com; 3 State Key Laboratory of Microbial Metabolism, School of Life Sciences & Biotechnology, Shanghai Jiao Tong University, Shanghai 200240, China

**Keywords:** cryptic gene cluster, dehydratase, heterologous expression, red/ET recombination

## Abstract

*Streptomyces thermolilacinus* SPC6 is a halotolerant strain isolated from the Linze Desert in China. It has a very high growth rate and short life cycle compared to other Streptomycetes, including the model organism *Streptomyces coelicolor.* The one strain–many compounds fermentation approach and global natural products investigation revealed that *Streptomyces thermolilacinus* SPC6 exhibits impressive productivity of secondary metabolites. Genome mining uncovered 20 typical secondary metabolic biosynthetic gene clusters (BGC), with a BGC *dmx* identified as completely silent. Subsequently, this cryptic BGC was successfully directly cloned and heterologously expressed in *Streptomyces* hosts, resulting in the discovery of a new lanthipeptide, dmxorosin. Notably, the proposed biosynthetic pathway indicates its potential as a basis for the synthetic biology of new lanthipeptide.

## 1. Introduction

To date, over 800 species of the bacterial genus *Streptomyces* have been identified. Classified within *Actinomycetota*, *Streptomyces* are characterized as Gram-positive and aerobic with high GC content genomes [1]. Since the 1950s, complex secondary metabolites have been isolated from *Streptomycetes*, many of which have been developed as antibiotics [2]. These antibiotics encompass a variety of structures, including aminoglycosides such as apramycin [3], nucleoside blasticidin [4], polyketide erythromycin [5], polyene nystatin [6], and polypeptide staphylomycin [7]. The increasing prevalence of multidrug-resistant bacteria due to widespread antibiotic use underscores the urgent need for novel antibiotic drugs [8,9]. Exploring new *Streptomyces* strains and their secondary metabolites holds great promise for advancing antibiotic drug development.

In recent years, genome sequencing has revealed that *Streptomyces* harbors a significantly larger number of secondary metabolism biosynthetic gene clusters (BGCs) than the number of identified natural products [10]. It suggests that numerous BGCs are either minimally expressed or completely inactive. Many of these “cryptic” BGCs are speculated to have the potential to produce novel compounds [11,12]. Various methods have been developed to activate these dormant gene clusters, including the manipulation of global transcriptional regulators [13], replacement of native promoters with more effective ones for structural genes [14], engineering of RNA polymerase or ribosomes [15], and the implementation of the “one strain many compounds” (OSMAC) strategy, which enables a single strain to produce different molecules under diverse growth conditions [16,17,18]. In cases where these approaches prove ineffective, isolating the biosynthetic gene clusters may be necessary, as well as expressing them in alternative hosts [19,20]. Various factors influence the selection of an appropriate heterologous host. It is advisable to adhere to the following guidelines to facilitate the identification of suitable candidates. First, the chosen heterologous hosts should be easily transformable and possess genetic tractability. Second, these hosts must have compatible genetic systems, including sigma factors and codon usages, that can effectively express the heterologous biosynthetic gene clusters (BGCs). Third, the heterologous hosts must provide a sufficient supply of biosynthetic precursors necessary for the production of heterologous natural products, preferably without the interference of competing pathways [21]. *Streptomyces* represents a significant genus of bacteria that is highly regarded for its capacity to produce natural products, accounting for nearly 70% of all isolated natural substances. Consequently, various species within this genus have been extensively employed as heterologous hosts [22].

**Figure 1:**
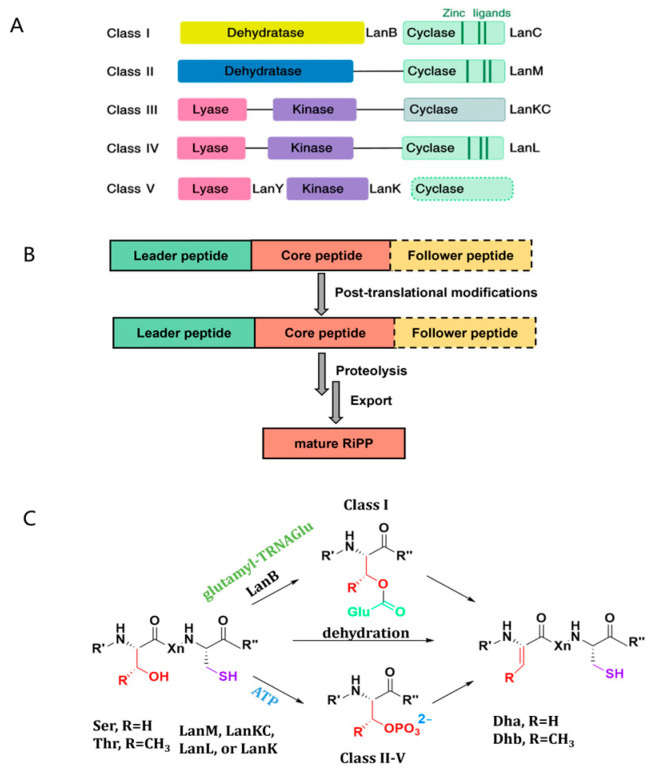
(**A**) General biosynthetic logic for RiPP, (**B**) five classes of lanthipeptide and its general biosynthetic genes, and (**C**) two dehydration patterns in the formation of Dha and Dhb amino acids. Dha: dehydroalanine; Dhb: dehydrobutyrine.

Ribosomally synthesized and post-translationally modified peptides (RiPPs) are widespread across all three domains of life, encompassing a substantial diversity of hidden chemicals with extensive biological functionalities [23,24,25]. RiPPs originate from ribosomally synthesized precursor peptides, typically characterized by a leader peptide at the N-terminus and a core peptide at the C-terminus (Figure 1A). The leader peptide is a crucial element for recognition by post-translation modifying enzymes. It is subsequently removed through proteolysis, while the core peptide serves as the site for post-translational modifications, culminating in the production of the mature product. In some instances, precursor peptides also include additional sequences at the C-terminus, known as follower peptides, which undergo proteolytic removal during RiPP maturation [26,27,28] (Figure 1A).

As one of the well-investigated families of RiPPs, the lanthipeptides are categorized into five classes based on the distinct biosynthetic mechanisms of the lanthionine (Lan) and methyllanthionine (MeLan) moieties (Figure 1B). Class I lanthipeptides undergo dehydration and cyclization reactions that are mediated by two separate enzymes: dehydratase LanB and cyclase LanC. In class II lanthipeptides, a bifunctional synthetase LanM, encompassing both a dehydratase domain and a cyclase domain, governs the dehydration and cyclization processes. While the dehydratase domain of LanM exhibits no sequence homology with other enzymes in databases, the cyclase domain signifies homology with the class I lanthipeptide cyclase LanC (Figure 1B). Class III and class IV lanthipeptides entail the dehydration and cyclization reactions, to be orchestrated by bifunctional synthetases LanKC and LanL, each harboring similar N-terminal phosphor-Ser or phosphor-Thr lyase domains and central kinase domains but differing in the C-terminal cyclization domains [29,30,31]. Recently, class V lanthipeptides have been identified, featuring a BGC lacking the conventional type I–IV lanthipeptide biosynthetic enzymes involved in Lan ring formation and instead employing a tri-enzyme cascade (Figure 1B) [32,33].

Lanthipeptides are distinguished by the presence of thioether cross-linked lanthionine Lan and MeLan [34,35]. These peptides, initially translated as precursor peptides known as LanA, contain an N-terminal leader peptide and a C-terminal core peptide. The precursor peptide is subject to post-translational modification (PTM) by enzymes and is subsequently excised following the installation of PTMs onto the core peptide [36,37]. The Ser or Thr residues are initially dehydrated with two chemical patterns. In the class I lanthipeptide, the Ser or Thr should be activated with glutamylation, which can be utilized by dehydratase LanB, leading to dehydroalanine (Dha) or dehydrobutyrine (Dhb) [38,39,40]. Otherwise, for other lanthipeptides’ (II–V) dehydration, the Ser or Thr are phosphorylated by ATP instead of glutamylation before enzymatic catalysis (Figure 1C). The cyclase catalyzes the intramolecular Michael-type addition of the thiol moiety of Cys onto the Dha or Dhb residues to generate Lan and MeLan rings in the core peptide [36,41]. Beyond the two PTMs, additional unusual modifications are sometimes carried out, such as incorporating D-amino acids, macrocyclization, N-acetylation, decarboxylation, etc., giving various lanthipeptides with diverse structures. Finally, the leader peptide is removed by proteolysis to provide the mature lanthipeptide [42,43,44]. The diverse array of continually discovered post-translational modification PTMs bestow modified peptides with enhanced metabolic stability and target specificity. Simultaneously, the ongoing exploration of novel PTM enzymes holds the potential to expedite the development of catalytic components applicable in synthetic biology and the synthetic tools utilized in medicinal chemistry.

In this study, the OSMAC fermentation strategy [45,46] revealed that *Streptomyces thermolilacinus* SPC6 could produce various compounds under different conditions. We examined the incomplete genome sequence of *Streptomyces thermolilacinus* SPC6 [47] and uncovered 20 typical secondary metabolic biosynthetic gene clusters (BGC). The gene cluster *dmx*, responsible for encoding putative lanthipeptide biosynthesis enzymes, was inactive, utilizing a gene knock-out approach. With the help of Red/ET cloning technology [48], we isolated the 23.5 kpb DNA fragment containing BGC *dmx* and expressed it in three different *Streptomyces* hosts. This process led to the discovery of a new lanthipeptide named dmxorosin, an unconventional class I lanthipeptide featuring two dehydratases in the biosynthesis. In addition to this new compound, identifying this biosynthetic pathway will be valuable for the synthetic biology of various lanthipeptide natural products.

## 2. Results

### 2.1. Secondary Metabolites Produced by S. thermolilacinus SPC6

**Figure 2:**
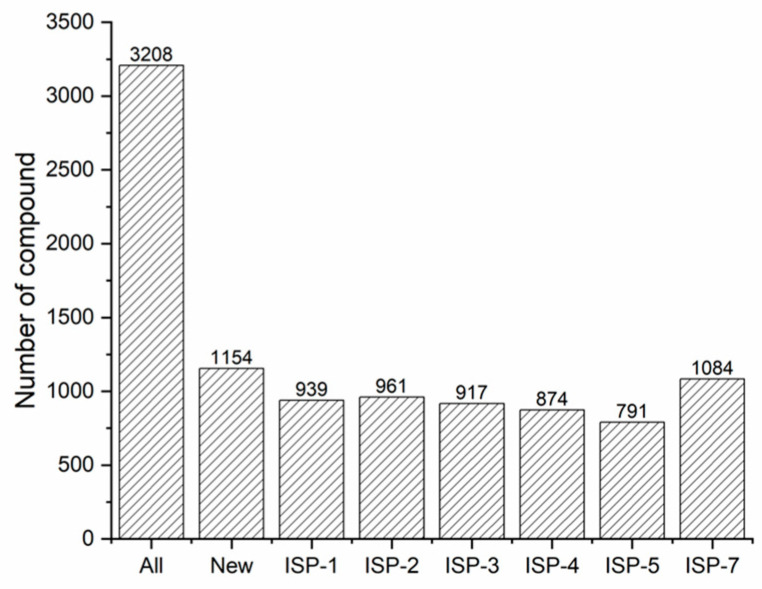
Secondary metabolite investigation of *S. thermolilacinus* SPC6. All: compounds from all six media together. New: the new compounds from all six media together. ISP-1, 2, 3, 4, 5, and 7: new compounds for the corresponding medium.

*S. thermolilacinus* SPC6 was incubated with six different media. The extracts of fermentation cultures were then analyzed using liquid chromatography–high-resolution mass spectrometry (LC-HRMS). Subsequently, the resulting data were subjected to the COCONUT database to identify known or unknown compounds based on the MS signals. The signals consistently present in all six extracts from the mediums were recognized as the desired signals. For the compound investigation, initially, a series of MS data in raw file format was transferred to mzXML format (https://npcompass.zju.edu.cn/zg-documents/ accessed on 16 January 2025). Then, the files were analyzed using the FUNEL algorithm. The handle well MS data were then linked to the COCONUT database to determine the known or unknown compounds (https://npcompass.zju.edu.cn/zh accessed on 16 January 2025). The output data are in CSV format (Table S4). The analysis data are arranged in the table by the mass spectral value and the signals’ retention time (RT). The value under each medium indicates the abundance of chemical elements in the corresponding fermentation culture. The value “−1” means undetectable. The columns “match” codes represent the PubChem library compounds. The “−1” means there is no match signal in the database and that it should be a new one. Upon meticulous examination of the MS values ranging from 100 to 2000, it was ascertained that *S. thermolilacinus* SPC6 yielded a minimum of 3208 compounds. Among these, 2054 are identified as known compounds, while 1154 remain unidentifiable in the COCONUT database. Notably, the incubation of *S. thermolilacinus* SPC6 with ISP7 medium produced the highest number of new compounds (Figure 2). The identified compounds spirofungin, kosinostatin, and BE-24566B, previously discovered and characterized in other *Streptomyces thermolilacinus* strains, have also been detected in *S. thermolilacinus* SPC6. The OSMAC strategy and PCA analysis have established that *S. thermolilacinus* SPC6 exhibits a robust capacity for producing secondary metabolites.

### 2.2. Genome Mining of S. thermolilacinus SPC6 for Putative Biosynthetic Gene Cluster

The complete genome sequence of *S. thermolilacinus* SPC6 is not available, but the DNA sequence of six contigs is Genbank: ASHX00000000.2. The antismash analysis identified 20 biosynthetic gene clusters (BGCs) in contig 1, while no secondary metabolite regions were found in the other contigs. *S. thermolilacinus* SPC6 contains two polyketide synthases (PKS), five non-ribosomal peptide synthases (NRPS), four ribosomally synthesized and post-translationally modified peptide (RiPP) synthases, and four terpenes synthases. The BGC responsible for biosynthesizing geosmin, ectoine, alkylresorcinol, flaviolin, and siderophore are all present in *S. thermolilacinus* SPC6, which are highly conserved in Streptomyces strains. Gene clusters encoding enzymes with low similarity to the known ones may potentially produce novel natural products. A detailed analysis of the BGCs is presented in Table S2. Among these, a class I lanthipeptide BGC, designated as 17, drew our interest. It contains a precursor gene, *dmxA*, two dehydratase enzyme genes, *dmxBL* and *dmxBS,* and a cyclase gene, *dmxC.* These genes together form a relatively complete cluster for the biosynthesis of lanthipeptides and are likely to yield new compounds. We have named this gene cluster “*dmx*” and intend to conduct further studies on it.

### 2.3. BGC dmx as a Silent Gene Cluster

To date, there have been no reports of the discovery of thiopeptide analogs in *Streptomyces thermolilacinus.* We carefully analyzed extracts from various fermentation cultures of *S. thermolilacinus* SPC6 strains using high-resolution MS and MS/MS in conjunction with the amino acid sequences of the core peptides of gene cluster *dmx*. We failed to identify one molecule that might be a product of BGC *dmx*. To further characterize whether gene cluster *dmx* is active, we knocked out the precursor peptide gene *dmxA* of the gene cluster using the in-frame deletion method (Figure S1). The wild-type and the mutant strain Δ*dmxA* were fermented using the same culture conditions. There was no obvious disappearance of peptide products when carefully analyzing the difference between the wild-type and mutant strains by LC-MS. The gene cluster *dmx* is indeed a “silent” gene cluster. To enhance support of this, we performed reverse transcriptase quantitative PCR (RT-qPCR) to analyze the transcription level of the gene cluster *dmx* in the heterologous strain Gsj02 and the wild-type strain *S. thermolilacinus* SPC6. Because the precursor gene *dmxA* is the key gene in biosynthesis and its transcription determines whether dmxorosin is produced, the RT-qPCR analysis of gen *dmxA* was performed. The primer for gene *dmxA* was qF_dmxA: GCCCCAGACCCTCGAACTC and qR_dmxA: ATGACGACCGTCGCGCAGA. The primer for reference 16s RNA genes was qF_16s: ACTCCTACGGGAGGCAGCA and qR_16s: ATTACCGCGGCTGCTGG. The relative expression level results indicated that the expression of *dmxA* in the strain Gsj02 was 365-fold higher than the wild-type. The data are shown in Figure S2. It confirmed that the low gene expression and the amount of object compound may be undetectable in the wild-type strain. All these data support our hypothesis that BGC dmx is “silent”.

### 2.4. Heterologous Expression dmx Producing a New Class I Lanthipeptide Dmxorosin

**Figure 3:**
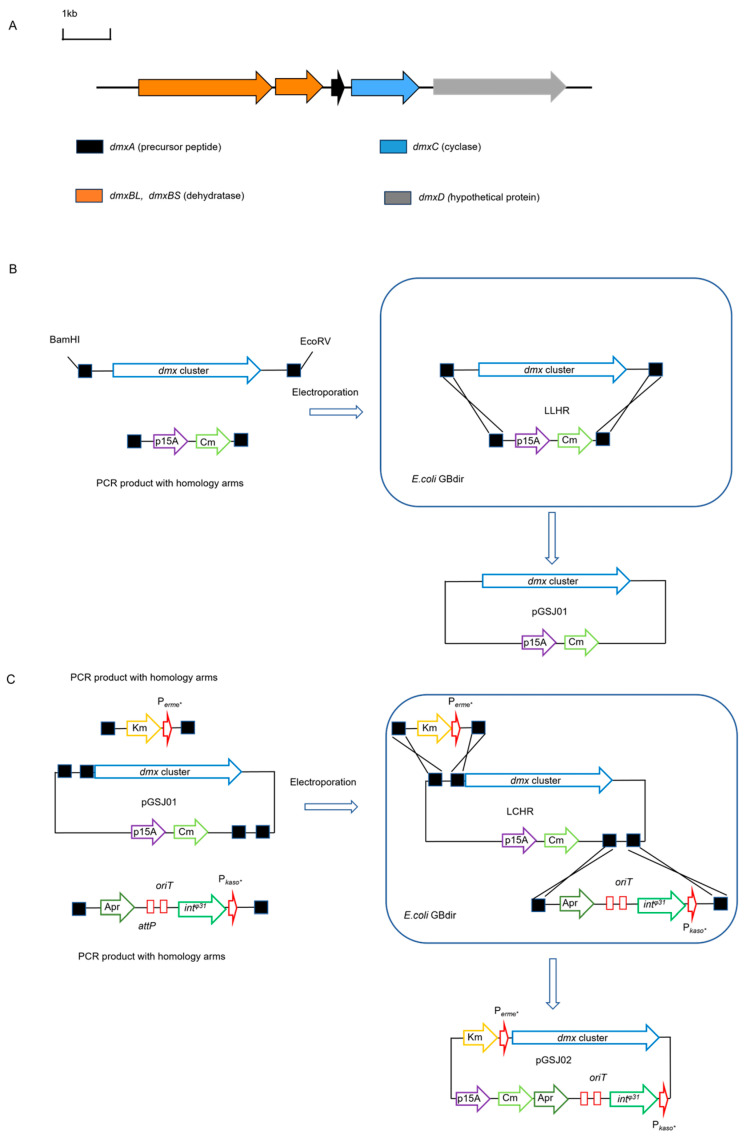
(**A**) The organizational structure of BCG *dmx*. (**B**) Linear plus linear Red/ET recombination (LLHR) in *E. coli* GBdir to construct pGSJ01 containing the BGC *dmx*. (**C**) Linear plus circular recombination (LCHR) in *E. coli* GBred to modify pGSJ01 to pGSJ02 containing conjugation, integration elements, and strong promoter. Black square: homology arm; p15A: origin in *E. coli*; Cm: chloramphenicol resistance; Km: kanamycin resistance; Apr: apramycin resistance; OriT: conjugation elements; int^φ31^: integration element; attP: integration sit; P_erme*_ and P_kaso*:_ promotor in *Streptomyces*.

**Figure 4:**
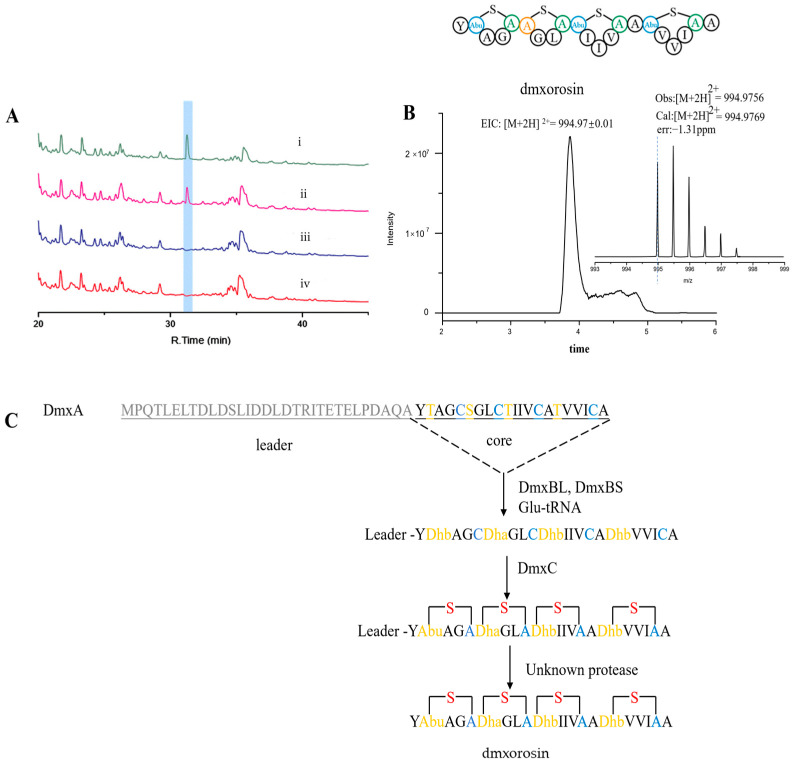
Production of dmxorosin: (**A**) HPLC analysis (i) pGSJ02 (a derivative of pSET152) containing BGC *dmx* expressed in *S. albus*, (ii) pGSJ02 (a derivative of pSET152) containing BGC *dmx* expressed in *S. albus* (parallel test), (iii) gene *dmxA* cloned in pSET152 and expressed in *S. albus*, and (iv) pSET152 in *S. albus*; (**B**) LC-HRMS analysis and structure of dmxorosin; (**C**) proposal of the biosynthetic pathway of dmxorosin. The leader peptide is shown in gray, and the core peptide is shown in black, except for Ser, Thr, and their dehydration residue, which are marked in orange, and the Cys and Ala residues are marked in blue. The sulfur atom from Cys is marked in red. The proteolytic cleavage sites are underlined. Abu: 2-aminobutyric acid.

The gene cluster *dmx* has been annotated to encode a pathway for RiPP. It differs from the typical class I lanthipeptide and belongs to a rare subtype featuring a split dehydratase LanB. The two distinct dehydratase enzymes are DmxBL and DmxBS. Furthermore, the *dmx* encompasses additional elements such as lanthipeptide cyclase gene *dmxC* and hypothetical protein gene *dmxD* (Figure 3A). The experimental results from the mutant studies showed that BGC *dmx* remained inactive in its native host. A 23.5 kpb DNA fragment was directly cloned using Red/ET technology and subsequently modified with elements for conjugation and site-specific recombination. The direct cloning and construction of the expression plasmid are shown in Figure 3B,C. Following this, the construct pGSJ02 was introduced into three *Streptomyces* hosts. The BGC dmx was successfully expressed in *Streptomyces albus* J1074 but not in *Streptomyces lividans* TK24 and *Streptomyces coelicolor* M1152 (Figure S4). This result showed that the *S. lividans* TK24 and *S. coelicolor* M1152 failed to meet the three principles for suitable heterologous expression hosts [21,49]. Fermentation culture analysis via HPLC revealed a new peak in *S. albu*s containing construct pGSJ02 but was absent in the strain with pSET152 as a negative control (Figure 4A). The LC-HRMS identified a peak with a two-protonated molecular ion at *m*/*z* = 994.9756 (cal. 994.9769), consistent with the HRMS of our deduction structure (hereafter named dmxorosin) (Figure 4B). The suggested molecule formula of dmxorosinis is C_88_H_141_N_21_O_23_S_4_. This compound is a derivative of the DmxA peptide containing lanthionine (Lan) and methyllanthionine (MeLan). In contrast to the non-ribosomal peptides, which typically have an average molecular weight of less than 1000 Da, RiPPs often exceed molecular weights of 2500 Da [50]. This larger size makes rapid analysis by NMR spectroscopy challenging, making mass spectrometry (MS) the most practical tool for characterizing the structures of RiPPs [51]. The mature product’s amino acid sequence or skeleton could be determined by the known amino acid sequences of precursor peptides, which are also verified by the tandem MS data [52,53]. For the class I lanthipeptide, the dehydratase and cyclase catalyze the formation of one or more cross-links between the threonine (T), serine (S), and cysteine (C). Therefore, combining tandem mass spectrometry of candidate compounds along with existing biosynthetic information can be an effective strategy for elucidating hypothetical structures of class I lanthipeptides. Thus far, many other research groups have also utilized such a strategy. According to the general biosynthetic mechanism, dehydration can only occur in threonine and serine. In Figure S5, the MS data of the fragmented ions b5, b9, b14, b15, b20, y6, y7, and y12 indicated that the three threonines (T2, T10, and T16) and serine (S6) of the core peptide were all dehydrated [51]. Then, we performed the NEM-derivative experiment. If dmxorosin has free cysteine, it will react with the NEM. None of the NEM-derivated products were detected (Figure S6), which indicated that the four cysteines were all Michael’s additions with double bonds of the Dha and Dha to form cross-links (Figure 1C). In class I lanthipeptide biosynthesis, the cyclase catalyzes the thioether cross-links between the dehydrated amino acid (Dha, Dhb) and cysteine (Figure 4C). Because the cycled products have the same molecular weight as the uncycled, the MS data can not distinguish them. Consequently, by integrating NEM experiments with random mass spectrometry techniques, we reasonably hypothesized the structure for dmxorosin (Figure 4B and Figure S5A).

The peptide DmxA consists of a leader peptide removed during maturation and a core peptide converted to dmxorosin. The dehydratases DmxBL and DmxBS are functional, like the N-terminal glutamylation and C-terminal elimination domains of full-length class I lanthipeptide dehydratase LanB. In dmxorosin PTM, DmxBL would utilize glutamyl-tRNA Glu as a co-substrate, and DmxBS would catalyze the glutamate elimination to generate the corresponding Dha/Dhb moieties. The BGC dmx also encodes a lanthipeptide cyclase, DmxC, forming the rings of the Lan and MeLan resides. A protease is not encoded in the *dmx,* and the mature lanthipeptide dmxorosin may be released from the precursor by an unknown protease in its heterologous host, *S. albus* (Figure 4C).

### 2.5. Antimicrobial Activity

The pure compound dmxorosin was tested for antibacterial properties. All tests showed no inhibition zones for Gram-positive and Gram-negative bacteria (Figure S7). Many newly discovered lanthipeptides have no antimicrobial activity but have antifungal, antiviral, antiallodynic, and morphogenetic functions [54,55,56]. The identified BGC *dmx* was utilized to conduct an extensive search of genomic databases for analogous BGCs in other organisms. Employing the DmxA sequence as a query, numerous homologous BGCs were identified, and subsequently, 1000 sequences were subjected to sequence similarity network (SSN) analysis. The SSN analysis unveiled that DmA is part of an extensive class encompassing SapT LanA (Figure S8). Dmxorosin was deduced to have a similar biological activity to lanthipeptide SapT [57], which exhibits morphogenetic activity. Limited by fermentation yields of dmxorosin, more compounds will be accumulated to explore potential biological function.

## 3. Discussion

Microorganisms produce secondary metabolites to adapt to environmental changes and competitive pressures [58]. These natural products are unnecessary during the cell growth phase and are usually produced when the cell stops growing or is under stress. They can help microorganisms resist external pressures, such as antibacterial, antifungal, antiviral, and anti-tumor activities [59]. *Streptomyces*, in extreme environments, usually uses special metabolic processes to inhabit unique ecological niches [60]. Microbial natural products continue to be regarded as a highly promising source of new pharmaceuticals [61]. As of 2009, over 22,000 microbial secondary metabolites had been documented [62]. *Streptomyces* has demonstrated a proven capability in generating novel bioactive secondary metabolites [63,64]. Nevertheless, there has been an increased re-isolation of the known compounds from previously studied *Streptomyces*, underscoring the necessity to identify, characterize, and scrutinize representatives of the new *Streptomyces* [65]. Additionally, it has become evident that unexplored and atypical environments, such as *Streptomyces* from uncharted ecosystems, potentially harbor significant bioactive metabolites with biotechnological applications [66].

The desert is one of the world’s largest ecosystems, covering around 30% of the land. Furthermore, 7% of deserts are hyper-arid, meaning that water systems are minimal. Arid areas are defined as biomes with a ratio of mean annual rainfall to mean annual evaporation of less than 0.05 and below 0.002 for extreme hyper-arid areas [67]. One of the least explored habitats is the Atacama Desert in northern Chile. It is known to be the driest desert on Earth, where conditions have been considered too extreme for any life to survive, with high levels of UV radiation, inorganic oxidants, areas of high salinity, and deficient concentrations of organic carbon. Despite such adversity, novel actinomycetes have been isolated and identified from this hyper-arid environment [68]. Extremophiles in the Qinghai-Tibet plateau soil and the Northwest China Desert show considerable diversity. Among them, many new *Streptomyces* strains have been discovered and proved to have the potential to produce bioactive compounds [69,70].

Silent BGCs, such as the *dmx* in *Streptomyces thermolilacinus* SPC6, are often considered evolutionary remnants of once-significant biosynthetic pathways. The BGC *dmx* may have originated from horizontal gene transfer, potentially providing advantages like antimicrobial activity in the past. However, decreased selective pressure may have led to its current inactivity. Alternatively, it could represent a recent acquisition that remains silent due to mutations or a lack of environmental stimuli [71]. This suggests the potential for future activation under specific conditions, such as stress or competition. Notably, although the BGC *dmx* is a silent and heterologous expression product, dmxorosin has no antimicrobial activity; its latent functions could involve roles in signaling pathways like quorum sensing, possibly leading to coordinated bacterial defense responses when certain thresholds are met [72,73].

*Streptomyces thermolilacinus* SPC6, a strain of streptomycete, was originally isolated from the Linze desert in China. This halotolerant organism is capable of thriving in a medium supplemented with NaCl within a concentration range of 0 M to 1 M. It demonstrates a rapid growth rate and possesses a comparatively abbreviated life cycle for a member of the streptomycete family. In the context of surface cultivation, the duration from spore germination to the formation of colonies harboring mature spore chains spans a mere 2 days at a temperature of 37 °C [47].To investigate its capability for producing secondary metabolites, *S. thermolilacinus SPC6* was fermented with six different media. The results showed that the ISP7 medium is the most suitable for natural compound production. By comparing the compound library, it was found that *S. thermolilacinus* SPC6 can produce 3208 compounds, among which 1154 are likely new chemicals. These numerous unknown compounds demonstrate that *S. thermolilacinus* SPC6 is a rich source of natural products and encourages further research for discovery. Bioinformatics analysis revealed that it contains 20 classical secondary metabolite biosynthetic gene clusters. Until now, no new compound has been discovered in *S. thermolilacinus SPC6.* A 23.5 kpb DNA fragment, including the intact silent BGC *dmx*, was cloned out using the Red/ET recombination and modified with genetic components for conjugation and site-specific recombination adapted for *Streptomyces*. A new lanthipeptide, dmxorosin, was discovered in the fermentation culture of the heterologous host *Streptomyce albus*. The MS analysis and NEM derivatization revealed that dmxorosin contains one lanthionine and three methyllanthionines. Dmxorosin, an unusual class I lanthipeptide with two split dehydratases in PTM, exhibits no apparent antibacterial activity. Its biological functions should be further investigated. Otherwise, according to the result of global secondary metabolites exploration, this halotolerant *Streptomyces* strain, SPC6, is worth continuing so as to mine other active natural products. Based on our discovery of the hidden natural product dmxorosin and its biosynthetic genes, combinatorial biosynthesis and synthetic biology approaches could produce various dmxorosin derivatives. New compounds could be screened for improved pharmaceutical characteristics, facilitating drug development.

## 4. Materials and Methods

### 4.1. OSMAC Strategy Fermentation and Natural Products Investigation

To produce secondary metabolites, the spore suspension of *S. thermolilacinus* SPC6 was inoculated into 5 mL of TSB medium and grown at 30 °C, 220 rpm for 48 h. The culture was used to inoculate 100 mL of several culture media (ISP1, ISP2, ISP3, ISP4, ISP5, and ISP7; formula listed in Table S3) [74] in a 500 mL flask at 28 °C and 220 rpm for 7 days. Then, the supernatant was collected by centrifugation at 6000 rpm for 10 min and extracted by isometric ethyl acetate. The precipitated mycelium was soaked in a tenfold volume of acetone for 6 h, and then the solution was vacuum-filtered with filter paper to discard the residue. All organic solvents were concentrated in a vacuum, and extractions from intracellular and extracellular were finally dissolved in 1 mL of methanol for the bioassay and liquid chromatography-mass spectrometry (LC-MS) analyses. The crude extractions were submitted to perform the LC-HRMS analysis using a Q-Exactive™ Focus Hybrid Quadrupole-Orbitrap Mass Spectrometer (Thermo Fisher Scientific Inc., Waltham, MA, USA) equipped with a Dionex Ultimate 3000 HPLC system (Thermo Fisher). We obtained six groups of first-order mass spectrometer (MS1) data. Then, all MS1 features of the six SPC6 fermentations were analyzed by the FUNEL algorithm. The FUNEL module encompasses the following steps: (1) the detection of features, (2) annotation of the isotopes and adducts/fragments, (3) alignment of the features across different samples, (4) filtering of the features using control samples, and (5) calculations of the exact mass based on the adducts/fragments for searches in the pen-access COCONUT compound database [75]. Subsequently, the InChIKeys of known compounds with identical exact masses are presented in the exported peak table [76]. The operation for this analysis was performed on the website https://npcompass.zju.edu.cn/zh (accessed on 20 July 2023).

### 4.2. Bioinformatic Analysis of Putative Secondary Metabolites BGC

In this study, the genome sequence of *S. thermolilacinus* SPC6 (Genbank: ASHX00000000.2) was submitted to The Antibiotics and Secondary Metabolite Analysis Shell Pipeline (antiSMASH) Server (https://antismash.secondarymetabolites.org/#!/start) (accessed on 15 January 2023) to analyze the biosynthetic gene cluster and predict the putative structure of the metabolites [77]. The parameter “detection strictness” was set as “relaxed” and the “Extra Features” were all on, including KnownClusterBlast, MIBiG cluster comparison, ClusterBlast, ActiveSiteFinder, TFBS analysis, Pfam-based GO term annotation, SubClusterBlast, RREFinder, and TIGRFam analysis.

### 4.3. ΔdmxA Mutant Strain Construction and qPCR Analysis

To inactivate BGC *dmx*, a 2 kpb upstream fragment and 2 kpb downstream fragment were amplified separately from the *S. thermolilacinus* SPC6 genomic DNA by PCR using the primer pair C17Left_For and C17Left_Rev, and the pair C17Right_For and C17Right_Rev, respectively (Table S1). The resulting fragments were purified using a kit from CWbio Co., Ltd. (Beijing, China) and then cloned into the *Hin*dIII/*Eco*RI site of pKC1139 using the ClonExpress II one-step cloning kit (Vazyme, Nanjing, China) to give the in-frame deletion construct pDMX01 (Figure S1). It was then transferred into *S. thermolilacinus* SPC6 via *E. coli-Streptomyces* conjugation. The BGC *dmx* in-frame deletion mutant (designated as *ΔdmxA*) was screened, and PCR and DNA sequencing confirmed its genotype. To perform reverse transcriptase quantitative PCR (RT-qPCR). The housekeeping 16 sRNA gene was used as a constitutive reference to normalize the gene expression of each target. RNA was retrieved from the strains for synthesizing the cDNA, grown under compound-producing conditions for 3 days. A total of 1 mg of total RNA was used and checked for the absence of genomic DNA by PCR. The Evo M-MLV RT Mix Kit (Accurate Biology, Changsha, China) was used for reverse transcriptation. The following qPCR analyses were performed in the CFX Manager thermocycler (Bio-Rad, Hercules, CA, USA) using 2× SG Green qPCR Mix (Bysbio, Changsha, China). The annealing temperature was set to 60 °C, and the 2-delta Ct method was employed to quantify the transcriptional level of the gene dmxA in the test strains [78].

### 4.4. Direct Cloning of BGC dmx and Heterologous Expression

The general procedure for direct cloning of cryptic BGC from genome DNA is shown in Figure S3. For BGC *dmx*, a linear DNA fragment, p15A-Cm, as a vector for direct cloning, was amplified by PCR with the primer B17DC (Table S1). The PCR product was purified by a DNA gel extraction kit (Fermentas) and dissolved in 20 μL of dH_2_O. A total of 50 μL of genomic DNA (~50–100 μg) was digested by *Bam*HI/*Eco*RV to release the DNA fragment containing the BGC *dmx*. After digestion, the DNA was precipitated with 3 M NaAc (pH 7.5) and 2.5 volumes of ethanol and finally re-dissolved in 20 μL of dH_2_O. A total of 1 μL of linear vector (PCR product) and 2 μL of digested genomic DNA were used for electroporation (a 1 mm electroporation cuvette, Eppendorf electroporator at 1300 V). A RecE/RecT-mediated linear plus linear homologous recombination (LLHR) happened in the *E. coli* GBdir to form the positive construct pGSJ01 (Figure 4B). Two linear DNA fragments, Apr-attp-OriT-int Φ31-P_kaso*_ and Km-P_erme*_ were obtained by PCR using primers B17oriT and B17erme (Table S1). After subsequent co-electroporation with pGSJ01, a Redαβγ-mediated linear plus circular homologous recombination (LCHR) occurred to introduce conjugation, integration elements, and strong promotor to form pGSJ02 (Figure 4C). After the failure in expression in *Escherichia coli*, pGSJ02 was transferred in the *Streptomyces* host, *S. albus* J1074, *S. lividans* TK24, and *S. coelicolor* M1152 via *E. coli-Streptomyces* conjugation, respectively. For the *E. coli-Streptomyces* conjugation, the pGSJ02 was transferred into *E. coli* ET12567 (pUZ8002). Then, this derivative was grown to an optical density at 600 nm of 0.3 to 0.4. Cells from a 20 mL culture were pelleted by centrifugation, washed with the same volume of LB broth twice, and re-suspended in 2 mL of LB broth as the *E. coli* donor. Spores of *S. albus* J1074, *S. lividans* TK24, and *S. coelicolor* M1152 (0.5 mL, 103–109/mL) stocked in 20% glycerol at −80 °C were washed twice with 0.5 mL of TES buffer (0.05 M, pH8.0), re-suspended in 0.5 mL of TES buffer, and then incubated for 10 min at 50 °C (heat shock) to activate the germination. The culture of spore suspension was incubated for 2–5 h at 37 °C after adding 0.5 mL of TSB broth. The cells were recovered and re-suspended in 0.5–1 mL of LB broth as the Streptomyces recipients. The donors (100 μL) and the recipients (100 μL) were mixed and spread equally onto ISP-7 plates freshly supplemented with 10 mM MgCl_2_. The plates were incubated at 28 °C for 20 h. After removal of most of the *E. coli* ET12567 donors by washing the plate surface with sterile water, the plates were overlaid with 3 mL of soft LB agar (0.7%) containing nalidixic acid (final concentration, 50 μg/mL) and antibiotic (for apramycin, 100 μg/mL). They were incubated at 28 °C until exconjugants appeared. The fermentation and compound isolation are the same as mentioned before.

### 4.5. Lanthipeptide Isolation and Structural Elucidation

The fermentation culture broth was centrifuged to yield a supernatant and a mycelium cake. The mycelium was abandoned because the target compound was discovered in the supernatant. The supernatant was extracted by an equal volume of ethyl acetate three times and evaporated to dryness. The crude extract was dissolved in MeOH and mixed with an appropriate amount of C18 powder for reverse-phase C18 column chromatography, eluted with a gradient elution of H_2_O: MeOH mixture from 70:30, 50:50, 30:70, and 10:90 to yield four fractions. The fractions were each analyzed by LC-MS to determine the target compound. Then, fraction B, containing dmxorosin, was purified by semi-preparative high-performance liquid chromatography (HPLC) with a linear gradient under the following program: (0–15 min, 5–40% ACN; 15–23 min, 40–100% ACN; 23–25 min, 100% ACN; 25–28 min, 5% ACN; 3.0 mL/min) to obtain dmxorosin (4 mg). HR-MS and tandem mass spectrometry (MS/MS) were used to determine the peptide’s amino acid sequence and putative rings in the peptide chain. An N-ethylmaleimide (NEM) assay was performed to detect a free sulfhydryl group in the peptides to verify the formation of thioether cross-links. A total of 100 μL dmxorosin solution with reduced TCEP was added to 10 μL of the NEM solution (50 mM in methanol) and incubated at 30 °C for 1 h before LC-MS analysis.

### 4.6. Bioassay

To investigate the antibacterial activity, Gram-negative bacteria *Pseudomonas aeruginosa*, *Escherichia coli* Bl21, and *Escherichia coli* K12, and Gram-positive bacteria *Kocuria rhizophila*, *Staphylococcus epidermidis*, and *Staphylococcus warneri* were chosen as indicators for the disk diffusion antimicrobial assay [79,80]. A total of 20 μL of different concentrations of dmxorosin (10 μg/mL, 50 μg/mL, and 100 μg/mL) was added to filter paper disks on agar plates that were preseeded with an overnight culture of the test strains at a concentration of 1% (*v*/*v*). A total of 10 μg/mL of kanamycin and solvent (70% ACN/30% H_2_O) were used as the positive and negative controls, respectively. After overnight incubation at 37 °C, the inhibition zone was measured using the crossover method, and the relative activities were indicated.

## Data Availability

The accession number of the draft genome sequence in GenBank is GCA_000478605.2.

## Supplementary Materials

The following supporting information can be downloaded at: https://www.mdpi.com/article/10.3390/ijms26041492/s1. References [48,74,81,82,83,84,85] are cited in the Supplementary Material.
