# Supplementary material for: Direct Cloning and Heterologous Expression of the Dmxorosin Biosynthetic Gene Cluster from Streptomyces thermolilacinus SPC6, a Halotolerant Actinomycete Isolated from the Desert in China
Source: Int J Mol Sci. 2025 Feb 11;26(4):1492. doi: 10.3390/ijms26041492 (PMC11855644; doi:10.3390/ijms26041492)
Supplement: Supplementary file 1 [file ijms-26-01492-s001.zip › ijms-3458587-supplementary.pdf]

International Journal of
Molecular Sciences

MDPI

Article

Direct Cloning and Heterologous Expression of the Dmxorosin Biosynthetic Gene Cluster from *Streptomyces thermolilacinus* SPC6, a Halotolerant Actinomycete Isolated from the Desert in China

Maoxing Dong [1], Huyuan Feng [1], Wei Zhang [2] and Wei Ding [3,*]

[1] School of Life Sciences, Lanzhou University, Lanzhou 730000, China; dongmx17@lzu.edu.cn (M.D.); fenghy@lzu.edu.cn (H.F.)
[2] Key Laboratory of Extreme Environmental Microbial Resources and Engineering of Gansu Province, Northwest Institute of Eco-Environment and Resources, Chinese Academy of Sciences, Lanzhou 730000, China; bestdw@163.com
[3] State Key Laboratory of Microbial Metabolism, School of Life Sciences & Biotechnology, Shanghai Jiao Tong University, Shanghai 200240, China
* Correspondence: weiding@sjtu.edu.cn

Supplementary Materials

Table S1. Strains, plasmids and primers used in this study.

Strain/Plasmid/Primer	Characteristic(s)	Reference
E.coli		
DH5α	Host for general cloning	Invitrogen
ET12567(pUZ8002)	Donor strain for conjugation between E. coli and *Streptomyces*	MacNeil et al 1992 [1]
GB2005	DH10B, *fhuA:: IS2, ΔrecET, ΔybcC*	Youming Zhang et al 2012 [2]
GB05dir	Red/ET recombination cloning strain (GB2005, *araC-BAD-ETgA*) The genes encoding the Redγ, RecE, RecT and RecA proteins were under the pBAD promoter and inserted into the original *ybcC* position	Youming Zhang et al 2012 [2]
GB05red	Red/ET recombination cloning strain (GB2005, *araC*-BAD-*αβγA*) The λ red operator and the gene encoding the RecA protein were inserted into the original *ybcC* position under the pBAD promoter	Youming Zhang et al 2012 [2]
Streptomyces		
S. thermolilacinus SPC6	Wild type strain	This study
S. albus J1074	Host for heterologous expression	Chater et al 1980 [3]
S. lividans TK24	Host for heterologous expression	Mary C. Betlach et al 1999 [4]
S. coelicolor M1152	Host for heterologous expression	Mervyn J Bibb et al 2014 [5]
Gsj01	BGC *dmx* in *S. albus J1074*, dmxorosin producing	This study
Gsj02	BGC *dmx* in *S. lividans TK24*	This study
Gsj03	BGC *dmx* in *S. coelicolor M1152*	This study
ΔdmxA	*dmxA* gene interruption in *S. thermolilacinus* SPC6	This study
**Plasmid**		
pKC1139	*E. coli*-Streptomyces shuttle vector for gene knockout	Hopwood, D.A. et al 2010 [6]
pSET152	*E. coli-Streptomyces* shuttle vector for integrative (phiC31) expression	Zixin Deng et

		al 2003 [7]
pDMX01	Inframe deletion of gene *dmxA* in *Streptomyces thermolilacinus* SPC6	
pGSJ01	BGC *dmx* cloned，p15A replicon，Cm^R	This study
pGSJ02	pGSJ-1 derivative with *oriT, AprR, phiC31*	This study
**Primer**		
B17DC_For	AAGCTTATCGCCCTGTCCATCGTCGGCACCGGCCTGGCGGCGGTGGGCGTCCTC GCCAACGGCACCGAGGAGCAGATCGGCACCTGCCCAGCCCGCCTAATGAGCG	This study
B17DC_Rev	GCGTCCTCGCCAACGGCACCGAGGAGCAGATCGGCACCTGCCCAGCCCGCCTA ATGRGCGAGCCTCCCAGTAGTACGACCCGCCATCGATTCTGAC-CTCCTGGTTATGTG	This study
B17oriT For	GTGCAGGACTGGGGGAGTTCAATTGTTCGTTAGGGTCTGTGCGCTGCCTACAAAG	This study
B17oriT Rev	TATCGATAAGCTTGATCTTAAGCTGGATGCCGACGGATTT	This study
B17erme For	GTGCAGGCAGTCCGCGAGCAGACGCTCGACCTGGAGCATATGTGGATCCTAC-CAACCGG	This study
B17erme Rev	CGGACATCACCTACAAGTGACGGTCCGGTCCGGCTGCCGACCCGCAC-CGGGCGGCGCCCG	This study
C**17**Left_F**or**	AAGAATTCCGGCGGGTGCGTGGTTCGTGGACG	This study
C**17**Left_**Rev**	GGTGGGCCCGCCTTTCGTGTCGTC	This study
C**17**Right_F**or**	CCGGACCCGCACCACAGGAGCCG	This study
C**17**Right_R**ev**	AAAAGCTTCGCGGCCAGCGCGCCGGGCGCGC	This study
C17V_For	CCCGGCTGTGGCATCCGCGCGAC	This study
C17V_Rev	CGCCGACGCTCGCCACTGTGTGG	This study

Table S2. Biosynthesis gene clusters for secondary metabolite in *Streptomyces thermolilacinus* SPC6 predicted by antiSMASH. The selected BGC 17 is highlighted in yellow

No	Type of BGC	Locus in genome	Most similar known cluster	Similarity
1	Type III PKS	1129,428 - 170,588	flaviolin/1,3,6,8-tetrahy-droxynaphthalene	100%
2	NRPS,lanthipeptide-class-ii	202,512 - 256,392	surugamide A/surugamide D	9%
3	Terpene, Type III PKS	301,739 - 344,754	alkylresorcinol	100%
4	Terpene	553,034 - 596,133	hopene	69%
5	NRPS	775,181 - 822,431	clipibicyclene/azabicyclene B/azabicyclene C/azabicyclene D	22%
6	NI-siderophore	890,307 - 925,431	speibonoxamine/desoxy-desferrioxamine D1/desferrioxamine D1/desferrioxamine B/didesoxy-desferrioxamine D1/desoxy-desferrioxamine B/didesoxy-desferrioxamine B	44%
7	RiPP-like	944,948 - 956,315	streptamidine	75%

8	RiPP-like	1,032,965 - 1,044,248	unknown	
9	Hydrogen-cyanide	1,091,268 - 1,104,442	aborycin	21%
10	Type I PKS,NRPS,NRPS-like	1,142,158 - 1,200,123	madurastatin D1/madurastatin D2/(-)-Madurastatin C1	4%
11	NRPS,Type I PKS,NRPS-like,HR-Type II PKS	1,208,057 - 1,381,741	colibrimycin	65%
12	Melanin	2,813,337 - 2,823,861	istamycin	11%
13	NRPS,betalactone	3,784,044 - 3,828,772	cystargolide A/cystargolide B	70%
14	NI-siderophore	4,251,471 - 4,281,264	desferrioxamin B/desferriox-amine E	100%
15	Ectoine	5,154,599 - 5,164,997	ectoine	100%
16	Phosphonate	5,691,173 - 5,722,670	rhizocticin A	21%
17	Lanthipeptide-class-i	5,833,368 - 5,858,811	akaeolide	12%
18	Thioamide-NRP	5,880,059 - 5,931,756	auroramycin	5%
19	Terpene	6,113,653 - 6,134,762	ikarugamycin	8%
20	Terpene	6,255,907 - 6,278,129	geosmin	100%

Table S3. The composition of the six media for fermentation.

Medium	Formula (1 L)
ISP1	Tryptone 5 g, Yeast extract 3 g, pH 7.0-7.2
ISP2	Yeast extract 4 g, Malt Extract 10 g, D-glucose 4 g, pH 7.3
ISP3	Oatmeal 20 g, Trace salt solution 1.0 mL, pH 7.2
ISP4	Soluble starch 10 g, K_2HPO_4 1 g, $MgSO_4$•$7H_2O$ 1 g, NaCl 1 g, $(NH_4)_2SO_4$ 2 g, $CaCO_3$ 2 g, Trace salt solution 1.0 mL, pH 7.0-7.4
ISP5	L-asparagine 1 g, Glycerol 10 g, K_2HPO_4 1 g, Trace salt solution 1.0 mL, pH 7.0-7.4
ISP7	Glycerol 15 g, L-tyrosine 0.5 g, L-asparagine 1 g, K_2HPO_4 0.5 g, $MgSO_4$•$7H_2O$ 0.5 g, NaCl 0.5 g, $FeSO_4$•$7H_2O$ 0.01 g, Trace salt solution 1.0 mL, pH 7.2-7.4

Table S4. Analysis results of the MS from the six fermentation cultures searched in the COCONUT compound database. The value "-1" means undetectable. The columns "match" codes represent the PubChem library compounds. The "-1" means there is no match signal in the database and it should be a new one. The details were supplied in an individual Excel file.

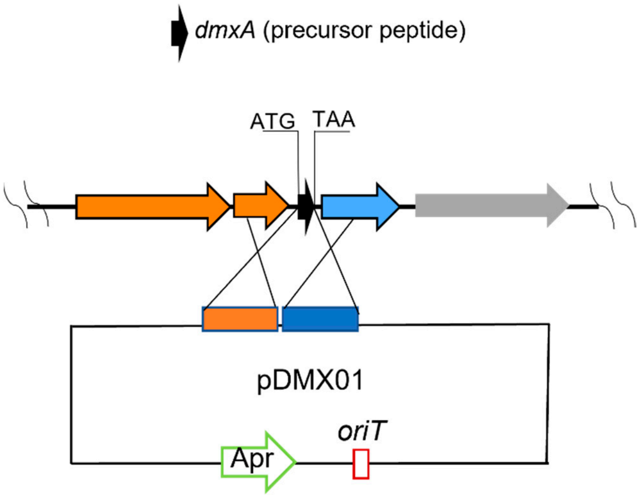

Figure S1. In-frame deletion of *dmxA* in *Streptomyces thermolilacinus* SPC6. Apr: apramycin; oriT: the origin of transfer. The orange box and blue box in pDMX01 indicate the homologous sequence of the genes in the chromosome. The crosswire hints that the homologous recombination.

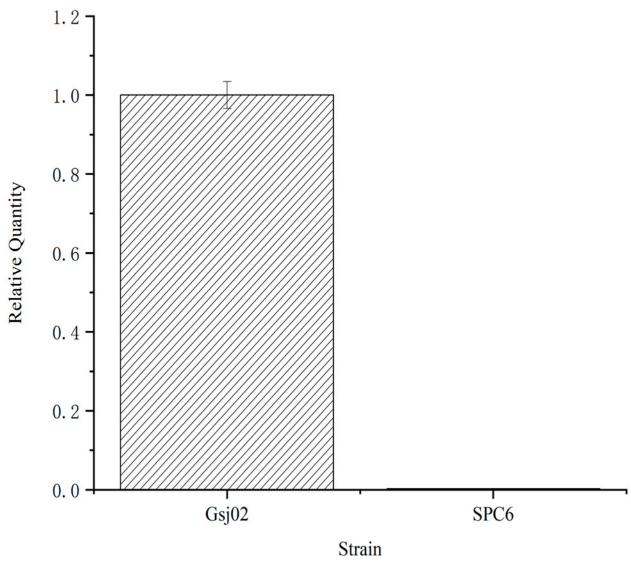

Figure S2. qPCR analysis of the transcription level of gene *dmxA* in the heterologous strain Gsj02 and wild-type strain *S. thermolilacinus SPC6*. The Y axis shows the relative transcription level of gene dmxA in the two strains.

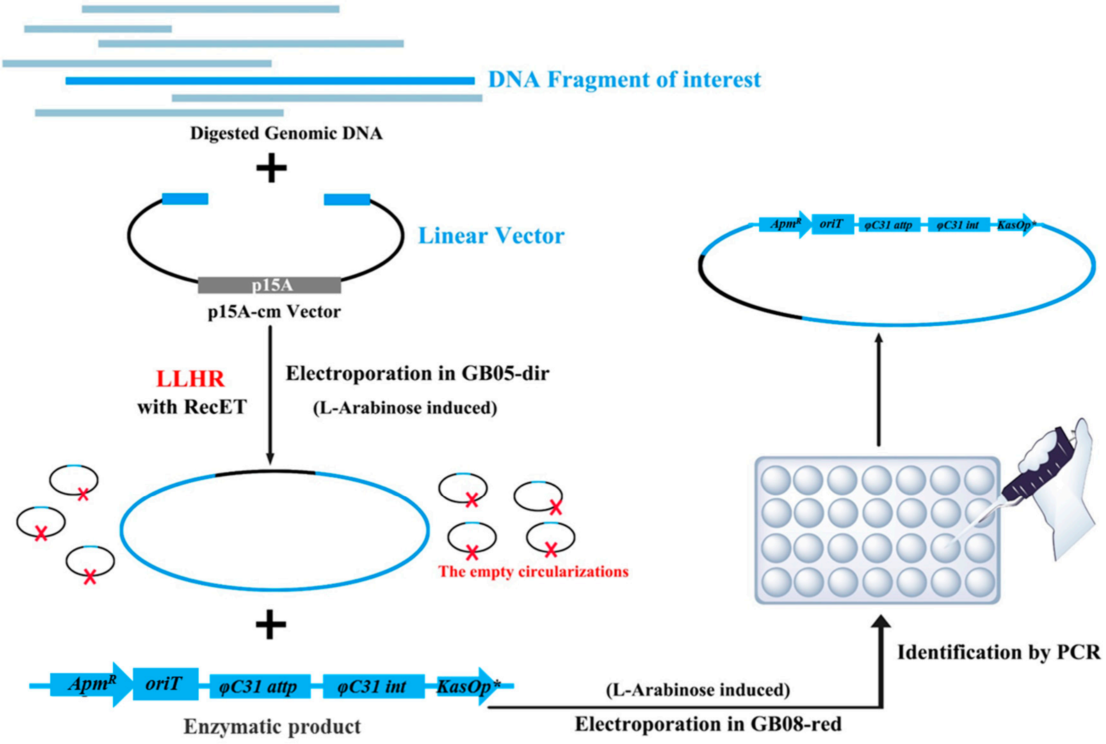

Figure S3. The general procedure for direct cloning of cryptic BGC from genome DNA.

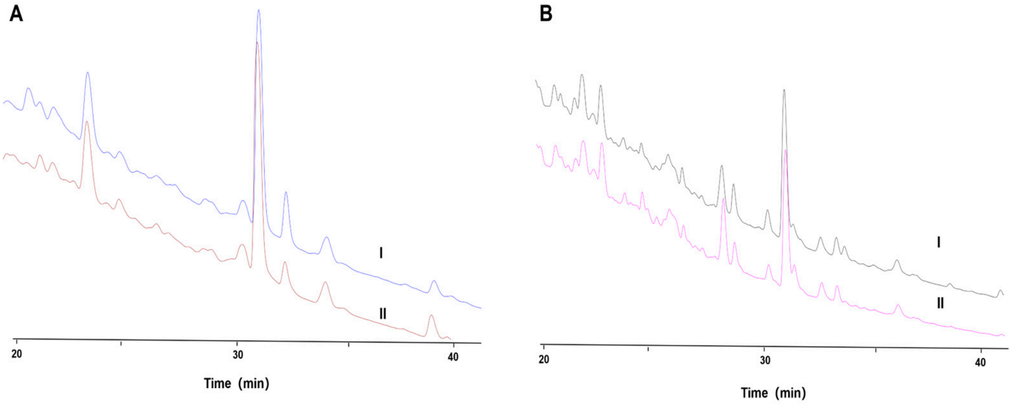

Figure S4. HPLC analysis of pGSJ02 expressed in *S. lividans* TK24 and *S. coelicolor* M1152. A: i) *dmxA* cloned in pSET152 and expressed in *S. lividans* TK24 as a control. ii) pGSJ02 expressed in *S. lividans* TK24; B: i) *dmxA* cloned in pSET152 and expressed in *S. coelicolor* M1152 as control. ii) pGSJ02 expressed in *S. coelicolor* M1152; Both hosts did not produce an obvious new peak, indicating the possibility of no expression of BGC *dmx*.

A

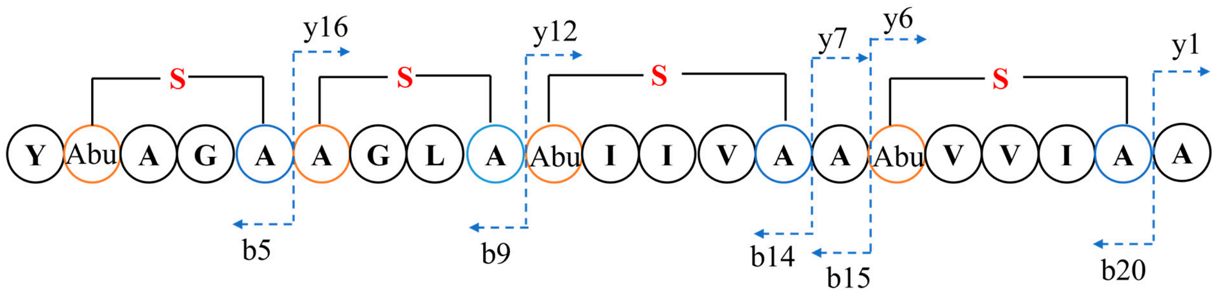

B

b ions	cal	obs	note	y ions	cal	obs	note
b5	478.1755	478.1742	-H_2O	y6	587.3221	587.3228	-H_2O
b9	820.3117	820.3071	-$2H_2O$	y7	658.3593	658.3560	-H_2O
b14	1331.5945	1331.5901	-$3H_2O$	y12	1169.6421	1169.6543	-$2H_2O$
b15	1402.6316	1402.6334	-$3H_2O$				
b20(z=2)	950.4530	950.4545	-$4H_2O$				

C

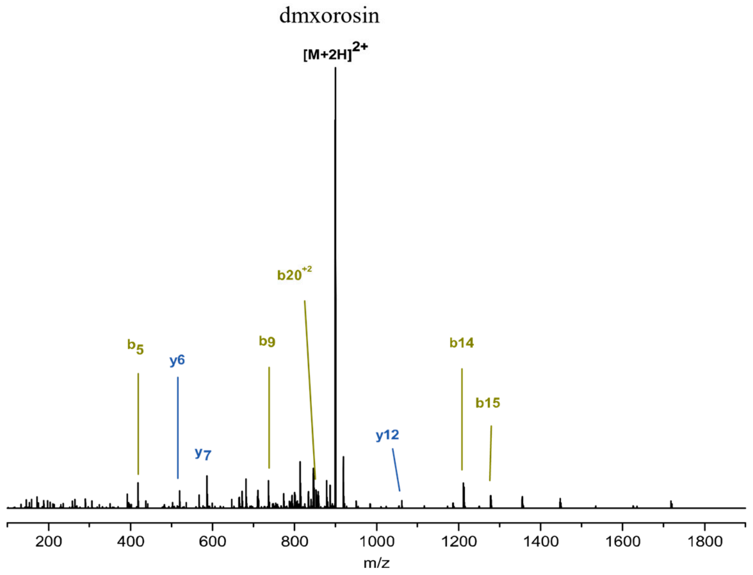

Figure S5. HR-MS/MS analysis of dmxorosin. A. Diagram representing the deduced structures of dmxorosin and the fragmentation pattern. B. A summary table of the characteristic fragment ions to map out the positions of the thioether linkage in dmxorosin. C. HR-MS/MS spectrum hinting the thioether linkage in dmxorosin.

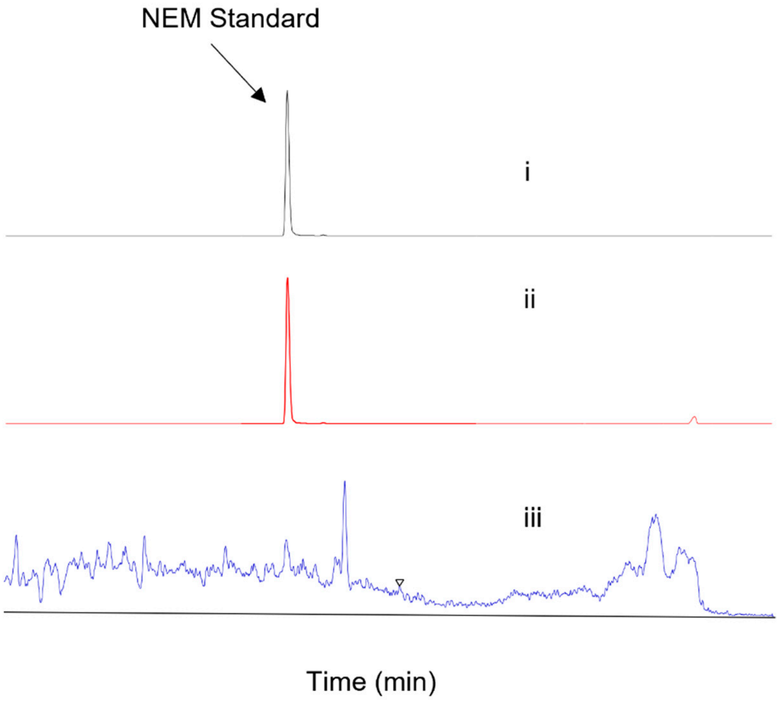

Figure S6. LC-HRMS analysis of the thioether rings in dmxorosin by NEM treatment. i) NEM +TCEP as control. EIC extraction of NEM; ii) NEM+TCEP+dmxorosin, EIC extraction of NEM; iii) NEM+TCEP+dmxorosin, EIC extraction of four NEM-dervied dmxorosin. No product.

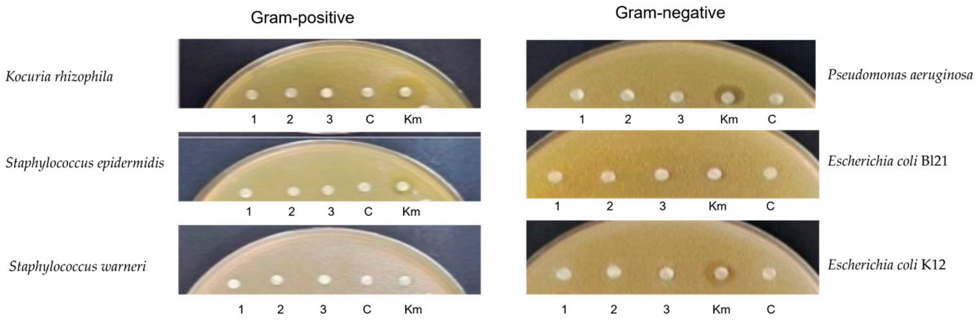

Figure S7. The disk diffusion test (Kirby-Bauer test) for antibacterial activity evaluation of compound dmxorosin. The left showed the results for Gram-negative bacteria *Pseudomonas aeruginosa, Escherichia coli Bl21, Escherichia coli K12* and the right are Gram-positive bacteria *Kocuria rhizophila, Staphylococcus epidermidis, Staphylococcus warneri.* The 1,2,3 represent the three concentrations of dmxorosin (10μg/ml, 50μg/ml, 100μg/ml), respectively. Km is kanamycin (10μg/ml) positive control. The C is bank control (70% ACN/30% H_2O).

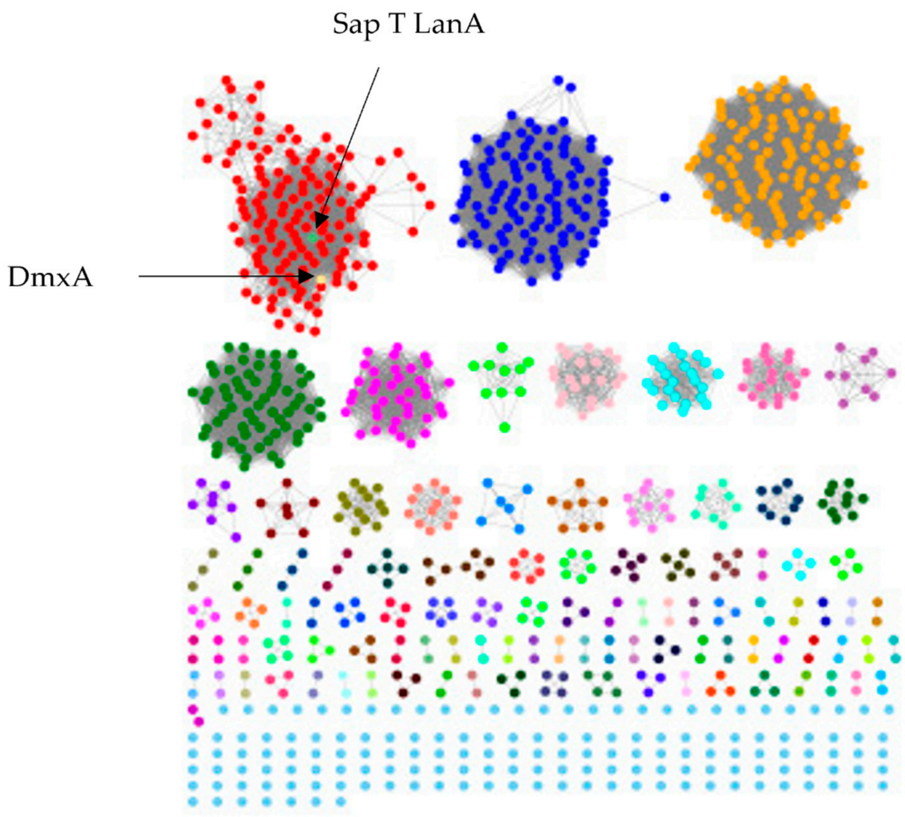

Figure S8. The sequence similarity network (SSN) analysis of DmxA and its homologs. The SSNs were generated based on the 1000 core peptide sequences with a threshold=10. Every node (circle) in the SSN corresponds to one unique precursor sequence. The dmxorosin precursor sequence (DmxA) is yellow compared to the SapT precursor peptide (SapT LanA) in green.

1. MacNeil, D.J., et al., *Analysis of Streptomyces avermitilis genes required for avermectin biosynthesis utilizing a novel integration vector*. 1992. **111**(1): p. 61-68.
2. Fu, J., et al., *Full-length RecE enhances linear-linear homologous recombination and facilitates direct cloning for bioprospecting*. 2012. **30**(5): p. 440-446.
3. Chater, K.F. and L.C.J.M. Wilde, *Streptomyces albus G mutants defective in the Sal GI restriction-modification system*. 1980. **116**(2): p. 323-334.
4. Ziermann, R. and M.C.J.B. Betlach, *Recombinant polyketide synthesis in Streptomyces: engineering of improved host strains*. 1999. **26**(1): p. 106-110.
5. Gomez-Escribano, J.P., M.J.J.o.I.M. Bibb, and Biotechnology, *Heterologous expression of natural product biosynthetic gene clusters in Streptomyces coelicolor: from genome mining to manipulation of biosynthetic pathways*. 2014. **41**(2): p. 425-431.
6. Aínsa, J.A.J.I.M., *Practical Streptomyces Genetics. T. Kieser, MJ Bibb, MJ Buttner, KF Chater, DA Hopwood*. 2010. **3**(4): p. 260-261.
7. Chen, S., et al., *Organizational and mutational analysis of a complete FR-008/candicidin gene cluster encoding a structurally related polyene complex*. 2003. **10**(11): p. 1065-1076.
